# Tackling emotional processing in adults with attention deficit hyperactivity disorder and attention deficit hyperactivity disorder + autism spectrum disorder using emotional and action verbal fluency tasks

**DOI:** 10.3389/fpsyt.2023.1098210

**Published:** 2023-02-01

**Authors:** Amélia Walter, Emilie Martz, Sébastien Weibel, Luisa Weiner

**Affiliations:** ^1^Institut des Neurosciences Cellulaires et Intégratives, Centre National de la Recherche Scientifique (UPR 3212), Strasbourg University, Strasbourg, France; ^2^Institut National de la Santé et de la Recherche Médicale U1114, Strasbourg, France; ^3^Department of Psychiatry, University Hospital of Strasbourg, Strasbourg, France; ^4^Laboratoire de Psychologie des Cognitions, University of Strasbourg, Strasbourg, France

**Keywords:** emotion dysregulation, verbal fluency, ADHD, ASD, neurodevelopmental disorders, executive functions

## Abstract

**Introduction:**

Attention Deficit Hyperactivity Disorder (ADHD) and Autism Spectrum Disorder (ASD) are two neurodevelopmental conditions with neuropsychological, social, emotional, and psychopathological similarities. Both are characterized by executive dysfunction, emotion dysregulation (ED), and psychiatric comorbidities. By focusing on emotions and embodied cognition, this study aims to improve the understanding of overlapping symptoms between ADHD and ASD through the use of verbal fluency tasks.

**Methods:**

Fifty-two adults with ADHD, 13 adults with ADHD + ASD and 24 neurotypical (NT) participants were recruited in this study. A neuropsychological evaluation, including different verbal fluency conditions (e.g. emotional and action), was proposed. Subjects also completed several self-report questionnaires, such as scales measuring symptoms of ED.

**Results:**

Compared to NT controls, adults with ADHD + ASD produced fewer anger-related emotions. Symptoms of emotion dysregulation were associated with an increased number of actions verbs and emotions produced in ADHD.

**Discussion:**

The association between affective language of adults with ADHD and symptoms of emotion dysregulation may reflect their social maladjustment. Moreover, the addition of ADHD + ASD conditions may reflect more severe affective dysfunction.

## 1. Introduction

Neurodevelopmental disorders affect 5–15% of children (1, 2) and often persist over the lifetime with significant impact on adaptive, academic, and social functioning (3). Attention Deficit Hyperactivity Disorder (ADHD) and Autism Spectrum Disorder (ASD) in particular are among the most common neurodevelopmental disorders (4).

Attention deficit hyperactivity disorder is characterized by a triad of symptoms: i.e., inattention, hyperactivity, and impulsivity. In children, the prevalence is estimated between 7.2 and 9.4% (5). Moreover, ADHD symptoms persist in adulthood in two-thirds of them (6, 7), as 4% of adults have ADHD (8–10). Nevertheless, the clinical presentation of ADHD in adults is different: i.e., motor hyperactivity symptoms are internalized in favor of mental hyperactivity, and attentional difficulties persist or even increase (11–14). In addition to these cognitive symptoms, adults with ADHD often report emotional difficulties. Indeed, emotion dysregulation (ED) concerns 30–70% of adults with ADHD (15). Emotion dysregulation can be defined by three dimensions: affect control, affective lability, and emotional over-reactivity (16–18). These symptoms can, respectively be reflected by irritability, frequent emotional fluctuations and greater emotional sensitivity. Importantly, neurodevelopmental and psychiatric comorbidities associated with ADHD are in part related to emotion dysregulation and its functional impact (19). Indeed, up to 75% of adults with ADHD have an associated psychiatric disorder (20), including depression in half the cases, anxiety disorders, bipolar disorder, and personality disorders (21). ADHD is also often associated with learning disabilities (22) and one in eight children have a co-occurring ASD (23).

Autism spectrum disorder is characterized by social communication peculiarities and the presence of restricted, repetitive behaviors (3). ASD affects approximately 1% of the world’s population (24). Despite obvious differences between diagnostic criteria of ADHD and ASD, the differential diagnosis can be challenging. Indeed, both conditions are characterized by attention difficulties (25, 26), disturbances in social interactions (27, 28), and abnormal sensorial sensitivity (29). Furthermore, emotion dysregulation is also common among autistic individuals, affecting up to 80% of them (30). No study to date has accurately described the facets of emotion dysregulation in adults with autism. That said, several studies highlight irritability, aggression, self-injury, impulsivity (31, 32) as manifestations of emotion dysregulation in children. Other studies have pointed out strong links between emotion dysregulation and other ASD symptoms such as anxiety (33), cognitive rigidity (34), and repetitive behaviors (35).

From a neuropsychological standpoint, emotional regulation is defined as the process of initiating, avoiding, inhibiting, maintaining, or modulating emotional states to accomplish individual goals or social adaptation (36). In neurodevelopmental conditions, emotion dysregulation is mainly explained by executive dysfunction (37, 38). Executive functions refer to a set of high-level cognitive processes that enable goal-directed behavior and problem solving (39). Executive functions can be classified into two categories: “cold” executive functions, such as mental flexibility, inhibition, and working memory, and “hot” executive functions, associated with the processing of social-emotional components (40, 41). These components include emotion regulation, empathy, rewards processing, and social adaptation (36, 37). Most studies focusing on the neuropsychological mechanisms of emotion dysregulation in neurodevelopmental disorders emphasize the involvement of cold executive functions. As an example, Barkley’s ADHD model (37) postulates that inhibition impairments cause emotional impulsivity (37, 42). In ADHD, emotion dysregulation also results from an inability to engage in self-regulatory actions, such as the ability to refocus attention (42). In ASD, inhibition and mental flexibility deficits are thought to result in perseverative behaviors, which in turn are related to maladaptive emotion regulation strategies, such as excessive rumination (43–46). When ADHD co-occurs with ASD, studies in children show that maladaptive emotion regulation strategies are exacerbated by ADHD symptoms (46). Therefore, the combination of these two conditions is characterized by more severe social and adaptive dysfunction compared to ADHD or ASD alone (47–49).

One way to study cold and hot executive functions is to use verbal fluency tasks (VFT). VFT, particularly letter and semantic conditions (i.e., generation of a maximum of words based on a letter or a semantic cue during a given time), have long been used to assess the executive and language functioning of patients (e.g., 50). VFT tap on lexical-semantic language processes (51), mental flexibility, inhibition, and information processing speed (52). These same processes are involved in the generation of emotional words (53). While the letter and semantic conditions preferentially target cold executive functions and semantic memory, respectively, the Emotional Word Fluency Test (EWFT), developed by Abeare et al. (54), assess the affective component of word production, i.e., affective language. Affective language corresponds to the production and comprehension of words or sentences with affective valence, but also emotional intonation and prosody (54). The EWFT consists of producing the maximum of emotion nouns during an allotted time (54). Disturbances in affective language have been found in several clinical populations, including individuals with right hemisphere brain damage (55, 56), depressive disorder (57), schizophrenia (58), or ASD (59). Nevertheless, in clinical settings, the EWFT has only been used in studies with individuals with traumatic brain injury (60). Using the EWFT with healthy subjects, Abeare et al. (61) found that trait anxiety was positively correlated with the number of emotions with negative valence produced. Furthermore, they showed that the physiological response of the sympathetic nervous system, e.g., skin conductance, was higher during emotional word generation compared to a control task, i.e., semantic VFT (i.e., “animal names”).

Typically, performances on VFT are assessed by the number of words generated, rule breaks, and repetitions (62). However, these quantitative measures provide little information on the cognitive mechanisms (i.e., language or executive) involved in word output. Several qualitative methods have therefore been developed to identify the cognitive processes involved in spontaneous word generation. For example, Troyer et al. (63) investigated the involvement of lexical-semantic processing and executive functions in VFT, *via* clustering and switching. Clustering corresponds to the production of words within a semantic or phonemic category, while switching reflects the ability to shift efficiently to a new category. Moreover, VFT are also influenced by parameters of imaginability, and concreteness, as well as linguistic parameters (e.g., lexical frequency). Indeed, access to the lexical stock is facilitated for more frequent and concrete words (64, 65). This facilitation allows a faster speed of word production, and an increased number of words produced for a specific time duration (66).

Few studies have investigated VFT performance in people with ADHD and autistic adults, and none have focused on affective language. For instance, adults with ADHD have been shown to produce fewer words than NT controls in semantic VFT (67, 68), and especially in letter conditions (69, 70). Since the letter conditions rely more on executive functions (71), these findings have been explained by the executive dysfunction usually associated with ADHD. In contrast, other studies found no deficits in VFT (72, 73). Therefore, the results are overall inconsistent in ADHD, probably due to the fact that only the number of words and errors were measured in most studies. In studies focusing on process-oriented measures of VFT (i.e., clustering and switching), some authors reported a decrease in the number of switches in the ADHD + ASD group population (68), while others have found the opposite pattern (73).

In ASD, studies have found reduced (74–76) or identical word count compared to control subjects in letter and semantic VFT (77–79). In contrast, the process-oriented measures of VFT, including clustering, switching, imaginability, and concreteness, did not differ from the NT controls (75, 77). These discrepant findings may be due to the heterogeneity of ASD presentations (80, 81). Indeed, autistic adults without intellectual disability have been found to produce fewer words during the first thirty seconds of letter and semantic conditions of VFT, which could be explained by an initiation deficit (82). Autistic children, on the other hand, generate less prototypical exemplars of semantic categories than NT controls (83), which can be explained by semantic deficits involved in comprehension and expression difficulties (84–86).

Regarding the assessment of hot executive functions using VFT, in ASD, most studies have focused on emotion identification, but not on affective word generation. Yet, emotional language disturbances involve both language comprehension and production (59). Autistic individuals produce sentences of greater duration, amplitude, and intensity than NT subjects (87). In addition, difficulties in identifying and describing one’s own feelings, i.e., alexithymia, are common in ASD (88). These difficulties are associated with the production of fewer emotional words when subjects are talking about negative events (89). Moreover, autistic adults assign less nuanced intensity and valence to stimuli compared to NT subjects (90, 91). For example, positive and negative images or words are evaluated less positively and negatively, respectively, than control subjects (92).

In ADHD, reduced abilities to recognize facial, vocal emotions (93, 94), and contextual information (95) have been reported (27, 96). In a study comparing children with ASD, ADHD, and ASD + ADHD, Tye et al. (97) found atypical processing of facial emotions for all the three groups. Specifically, autistic children displayed reduced neurophysiological response to angry faces in early stages of information processing. In contrast, children with ADHD displayed electrophysiological abnormality at the contextual processing stage when they were confronted with the emotions of fear and joy. The ADHD + ASD group presented an additive effect of the unique deficits of both conditions. Therefore, emotion processing seems to differ between the two groups, with autistic individuals presenting more difficulties at the early stages of processing of anger, whereas children with ADHD have difficulties at later stages of processing of fear and joy.

In addition to EWFT, other VFT involving embodied cognition may provide additional information about emotional processing in ADHD and ASD. Embodied cognition refers to the relationship between cognitive and bodily experience, which can be tackled *via* the VFT condition of action words generation (98, 99). In this task condition, subjects have to produce as many names of action verbs as possible during an allotted time. Whereas other VFT (i.e., semantic and letter) involve mainly temporo-parietal regions (100), Action Verb Fluency Test (AVFT) is sensitive to the activation of left fronto-striatal circuits (101). Fronto-striatal circuits are involved in “hot” executive functions such as rewards processing, emotion regulation and motivational states (102), and dysfunction of these circuits is involved in the cognitive and motivational control impairment found in ADHD (103). Relatedly, the same regions are thought to be involved in the difficulties in predicting emotions and actions of others in ASD (104). When using AVFT with autistic adults, Inokuchi and Kamio (105) found a reduced production of semantic clusters and an increased production of phonological clusters compared to control subjects, which they attributed to initiation difficulties due to executive dysfunction (106). However, AVFT performance has not been investigated in ADHD.

To our knowledge, no study has compared the performance on the EWFT and the AVFT in adults with ADHD and ADHD + ASD. Yet, it is of the upmost importance to better understand the overlap between these two conditions, especially regarding emotional processing, given its impact on quality of life and social functioning. The present study aims to evaluate several types of VFT, including EWFT and AVFT, in adults with ADHD, ASD + ADHD, and NT controls. We are particularly interested in the relationship between emotion dysregulation and verbal fluency tasks performance on EWFT and AVFT in ADHD. To this end, the number of words and errors produced, but also the type, the valence, the arousal, and the dominance of words generated in the EWFT and the AVFT will be analyzed. Given that emotion dysregulation is found in ADHD and ASD, we hypothesize that affective language particularities, i.e., a reduction in the number of emotions produced on the EWFT, will be found both in ADHD, and ADHD + ASD compared to NT controls. However, since impairments at earlier stages of emotion identification are found in ASD (97) compared to ADHD (89, 93), we expect adults with ADHD + ASD to produce fewer emotions on the EWFT compared to adults with ADHD. Due to abnormalities in the contextual processing stages of specific emotions (97), we also hypothesize that the emotion generation difficulties of adults with ADHD will be related to joy and fear. In view of results from a previous study on co-occurring ADHD + ASD (97), this group may present with the emotional particularities of both conditions with emotion generation difficulties related to joy, fear and anger. Regarding AVFT, given the primary deficits in predicting, and interpreting the actions of others (107), and motor issues involving fronto-striatal circuits (108) in ASD, adults with ADHD + ASD are expected to produce fewer and rarer (low frequency in the language) action verbs than adults with ADHD and NT controls, whereas adults with ADHD will produce fewer action verbs than NT adults. Nevertheless, due to the hyperactivity found in ADHD, the action verbs produced are expected to be more arousing than those found in NT.

## 2. Materials and methods

### 2.1. Participants

Fifty-two adults with ADHD aged 18–57 (M = 34.75; SD = 11.49), and 13 adults with co-occurring ADHD + ASD aged 19–67 (M = 32.15; SD = 13.95) without intellectual disability, communication difficulties, nor difference in gender distribution (see Table 1) were recruited from the University Hospital of Strasbourg. We were also interested in a group of 12 autistic adults aged 18–48 (M = 26.92; SD = 8.35) from another study on emotion dysregulation conducted by our team. After diagnoses of ADHD and ASD were established by senior psychiatrists according to DSM-5 criteria (3), subjects were offered participation in one of the two studies. These diagnostic interviews also involve establishing a differential diagnosis between ASD and ADHD. Among the ADHD group, 78% of subjects had combined ADHD presentation, 20% of them had inattentive subtype, and the last 2% had hyperactive presentation. Subjects with psychopathological disorders, i.e., depressive disorders and anxiety, were excluded in the ADHD group but not in the ASD and ADHD + ASD group. Ten subjects from the ASD group had experienced one or more past depressive episodes. Four of them also suffered from anxiety disorders. In the ADHD + ASD group, four adults had depression, whereas three had an anxiety disorder. In addition, one participant had bipolar disorder. Twenty-four age-matched NT controls aged 20–46 (M = 32.71; SD = 9.45) were recruited. These participants did not have any history of neurological, psychiatric, or substance use disorders and did not use any psychotropic medication. Adults with ADHD and ADHD + ASD had significantly higher ADHD symptoms (measured by the Wender-Reimherr Adult Attention Deficit Disorder Scale; (16), compared to NT controls (*p* < 0.001; Table 1). In contrast, the Autism Spectrum Quotient score (AQ–10 items); (109) and social adjustment difficulties (WRAADDS); (16) were higher in the ADHD + ASD group compared to the ADHD group (*p* = 0.011; *p* = 0.010, respectively) and to NT subjects (*p* < 0.001). All participants were native French speakers. This study was approved by ethics committees (CPP South Mediterranean II; IDRCB: 2017-A01618-45 and CPP East of France; No. SI 21.01.21.41923).

**TABLE 1:** Demographic, clinical and neuropsychological characteristics of participants in each group.

	ADHD (AD) (*N* = 52)	ADHD + ASD (AD + AS) (*N* = 13)	Neurotypical controls (NT) (*N* = 24)	Statistical analyses	*P*	*Post hoc*
**Demographic characteristics**						
Age	34.75 (11.49)	32.15 (13.95)	32.71 (9.45)	χ^2^ (2) = 1.699	0.428	/
Gender	46.14% F	38.46% F	66.66% F	χ^2^ (2) = 3.452	0.178	/
Years of education	13.61 (3.29)	15.09 (1.81)	15.37 (1.53)	χ^2^ (2) = 5.764	0.056	/
IQ estimation	111.19 (14.15)	108.55 (15.65)	105.00 (9.52)	F (2,65) = 1.635	0.203	/
WAIS-III–vocabulary (SS)	12.12 (2.74)	13.00 (2.00)	10.71 (1.60)	χ^2^ (2) = 6.250	**0.044**	AD + AS > NT
**Clinical characteristics**						
WRAADDS—inattention	17.71 (3.85)	17.17 (3.92)	5.74 (4.34)	F (2,61) = 63.991	<0.001*	AD, AD + AS > NT
WRAADDS—hyperactivity	7.54 (3.24)	7.33 (1.86)	2.52 (2.39)	χ^2^ (2) = 25.138	<0.001*	AD, AD, AD + AS > NT
WRAADDS—impulsivity	12.31 (3.77)	10.50 (5.32)	4.00 (3.33)	χ^2^ (2) = 32.145	<0.001*	AD, AD + AS > NT
WRAADDS—affective Lability	11.69 (2.92)	11.33 (4.55)	6.00 (4.17)	χ^2^ (2) = 22.063	<0.001*	AD, AD + AS > NT
WRAADDS—emotional over-reactivity	12.80 (3.16)	13.33 (3.20)	5.13 (3.33)	χ^2^ (2) = 34.122	<0.001*	AD, AD + AS > NT
WRAADDS—social maladjustment	21.83 (8.42)	32.33 (8.71)	6.26 (6.67)	F (2,61) = 39.442	<0.001*	AD + AS > AD > NT
Autism spectrum quotient	4.29 (1.79)	6.50 (2.37)	2.87 (2.32)	F (2,64) = 12.801	<0.001*	AD + AS > AD > NT
BDI—total	19.80 (10.64)	29.92 (18.28)	6.37 (5.95)	χ^2^ (2) = 25.780	<0.001*	AD, AD + AS > NT
GAD-7—total	9.29 (4.54)	5.80 (6.22)	2.79 (1.76)	χ^2^ (2) = 18.738	<0.001*	AD > NT
TEMPS-A—cyclothymic temperament	22.28 (7.98)	17.67 (10.31)	7.35 (5.80)	F (2,60) = 27.276	<0.001*	AD, AD + AS > NT
**Neuropsychological characteristics**						
VFT free (number of words)	69.03 (22.27)	54.91 (17.66)	73.54 (19.50)	F (2,64) = 1.698	0.191	/
VFT letter (number of words)	28.46 (6.95)	26.45 (7.02)	29.92 (6.91)	F (2,64) = 0.635	0.533	/
VFT semantic (number of words)	37.09 (8.04)	32.36 (8.22)	38.50 (6.28)	F (2,64) = 1.136	0.328	/
TMT Part B minus Part A (seconds)	36.52 (18.35)	24.82 (15.53)	30.41 (14.45)	χ^2^ (2) = 2.479	0.290	/
Stroop interference minus reading (sec)	34.37 (14.27)	29.02 (7.03)	25.83 (8.39)	χ^2^ (2) = 5.368	0.068	/
Digit span forward (number of digits)	6.31 (1.16)	5.83 (0.41)	6.67 (0.76)	χ^2^ (2) = 4.533	0.104	/
Digit span backward (number of digits)	5.20 (1.43)	4.67 (0.52)	5.79 (1.47)	χ^2^ (2) = 3.868	0.145	/
Hayling test inhibition (seconds)	60.04 (32.29)	57.47 (13.84)	63.73 (33.93)	χ^2^ (2) = 0.240	0.887	/
Hayling test inhibition (errors)	6.94 (3.73)	6.33 (3.33)	4.42 (2.43)	χ^2^ (2) = 6.708	0.035*	AD > NT
TAP Go-Nogo 1 out of 2 (errors)	0.97 (0.98)	1.17 (1.94)	0.75 (0.94)	χ^2^ (2) = 1.114	0.573	/
TAP Go-Nogo 2 out of 5 (errors)	0.34 (0.72)	0.17 (0.41)	0.04 (0.20)	χ^2^ (2) = 3.925	0.140	/
ToL (total correct)	4.86 (2.38)	4.33 (2.73)	4.25 (2.64)	F (2,62) = 0.919	0.640	/
ToL (additional movements)	26.80 (17.96)	28.33 (14.83)	28.46 (18.23)	χ^2^ (2) = 0.195	0.907	/
ToL (initation time)	73.30 (47.24)	56.16 (25.97)	61.29 (44.97)	χ^2^ (2) = 1.698	0.428	/

*p < 0.05; Years of education, number of years after the first grade in elementary school; SS, scaled score. *p*-values in bold = significant *post hoc* comparisons.

### 2.2. Materials and procedures

All subjects, with the exception of the ASD group, participated in a neuropsychological assessment and completed self-report questionnaires. Autistic adults only completed the VFT conditions.

#### 2.2.1. Questionnaires

Attention deficit hyperactivity disorder and ASD symptoms were, respectively measured by the Wender-Reimherr Adult Attention Deficit Disorder Scale (WRAADDS); (16) and the Autism spectrum Quotient (AQ–10 items); (109). The WRAADDS is a self-reported scale assessing the three core symptoms of ADHD, some dimensions related to emotion dysregulation (e.g., affective lability and emotional over-reactivity), as well as other areas frequently disrupted in ADHD, such as organization, academic problems and social adjustment. The 10-item Autism Spectrum Quotient is a self-administered questionnaire used to assess traits of autism in adults without intellectual disability. Items are related to domains of the dyad of impairments, i.e., social communication peculiarities (e.g., I find it easy to “read between the lines” when someone is talking to me), restricted and repetitive behaviors (i.e., I like to collect information about categories of things).

Depressive and anxiety symptoms were measured by the Beck Depression Inventory (BDI); (110), and the Generalized Anxiety Disorder assessment 7-item (GAD-7); (111). These screening tools are used to measure the severity of depression and generalized anxiety in normal and psychiatric populations.

Emotion dysregulation was evaluated by the cyclothymic dimension of the Temperament Evaluation of Memphis, Pisa, Paris and San Diego-autoquestionnaire version (TEMPS-A); (112) and the WRAADDS (16). The TEMPS-A is a questionnaire designed to measure affective temperaments with four subscales: cyclothymic (e.g., my ability to think varies greatly from sharp to dull for no apparent reason), irritable (e.g., people tell me I blow up out of nowhere), hyperthymic (e.g., I love to tackle new projects, even if risky), and anxious (e.g., when someone is late coming home, I fear they may have had an accident). To study ED, in particular emotional lability in ADHD, we were interested in the cyclothymic temperament subscale of the TEMPS-A. All the scales used were self-reported and validated in French (113–117) with Likert scales to consider the frequency of symptoms.

#### 2.2.2. Neuropsychological assessment

Intelligence quotient: In ADHD, ADHD + ASD, and NT groups, IQ was estimated *via* the matrix and the vocabulary subtests of the Wechsler Adult Intelligence Scale–Third Edition (WAIS-III); (118), whereas the matrix and information subtests of the WAIS-IV (119) were used to estimate IQ in the ASD group.

Executive functions: verbal working memory was assessed by the digit-span task (WAIS-III); (108). The “Go-Nogo” subtests with 1-target and 2-target from the TAP 2.3 (120) and the Stroop task (D-KEFS); (121) were, respectively used to investigate motor inhibition and interference inhibition. In addition, the Hayling test (122) was administrated to evaluate semantic inhibition abilities. The “Flexibility” condition of the Stroop task and the Trail Making Test (TMT A&B); (123) has been proposed to measure, respectively cognitive flexibility, speed and attention switching. The Tower of London test (124) was administrated to analyse planning strategies. In addition to EWFT and AVFT, three other conditions of VFT were used, i.e., the free word generation condition (125), as well as the semantic and letter conditions (62).

#### 2.2.3. EWFT and AVFT

Emotional Word Fluency Test (EWFT); (54): the EWFT measures the production of emotion words within 1 min. Similar to other VFT, three scores are generated, including the number of emotion words, the number of repetitions, and the number of rule breaks (i.e., non-emotion words). Feelings such as “love,” or typical adjectives describing an emotion such as “happy” were included. In order to measure the variety of the emotional lexicon, words were systematically categorized into one of the six basic emotions defined by Ekman (126). We used Plutchik’s wheel of emotions (127), which is based on primary emotions and associated secondary ones. A percentage of the number of words generated by the subject related to each primary emotion (see Table 2) was calculated to compare the type of emotion. For example, the percentage of words related to joy compared to the total number of words produced was reported. We were also interested in the frequency of occurrence of every emotions produced in the whole sample. We then measured the average frequency of the emotions generated by each subject. This measure allows to evaluate the ability to produce more varied and less common emotions.

**TABLE 2:** Examples of words related to the six primary emotions defined by Ekman (126) and Plutchik (127).

Joy	Fear	Anger	Sadness	Surprise	Disgust
Joy	Fear	Anger	Sadness	Surprise	Disgust
Love	Anxiety	Envy	Melancholy	Astonishment	–
Happiness	Anguish	Hate	Depressed	–	–
Excitement	Jealousy	Indignation	Despair	–	–
Euphoria	Stress	Rage	Bitterness	–	–
Pleasure	Panic	Fury	–	–	–

Action Verb Fluency Test (AVFT); (101): This task measures the production of action verbs within 1 min. The number of action nouns, repetitions, and rule violations (i.e., non-action words, such as “appear”) were calculated. The frequency of occurrence of action verbs was also averaged over the entire sample. Repetitions and errors were not analysed because their occurrences were too rare in the total sample.

Valence, arousal, dominance: Based on Osgood et al. (128), we analysed the valence, the arousal and the dominance of words. The valence refers to the pleasantness/unpleasantness of a word, while arousal refers to the intensity of the emotion provoked by the stimulus (129, 130). Dominance is the subjectively measured degree of control exerted by a word. This dimension expresses the extent to which the word denotes something that is weak/submissive or strong/dominant (130). This method, previously used in the analysis of the EWFT (61) and the AVFT (131), corresponds to study the average valence, arousal, and dominance of words generated by using a corpus of English words (130). This corpus established for a total of 13,915 words allows quantifying these variables for the majority of emotional and action words evoked. For each word, a score for valence, arousal and dominance is available based on the ratings of one million subjects. Specifically, ratings on (i) the valence of each word ranging from unhappy to happy; (ii) arousal from excited to calm and (iii) dominance from in control to controlled are available (130). The French words generated in the VFT were translated and then back-translated with translation dictionaries, i.e., WordReference and Linguee. Translations were chosen according to previously generated words when several equivalents existed.

### 2.3. Statistical analyses

Percentages for qualitative variables and means as well as standard deviations for quantitative variables were included in descriptive statistics. One-way ANOVA and *post hoc* Tukey were conducted to compare demographic and clinical variables between groups. One-way ANOVA and *post hoc* Tukey were also used for each VFT variables (i.e, quantity of words, errors, repetitions, valence, arousal, dominance, and percentage for each primary emotion). Assumptions for ANOVA were verified by the Shapiro-Wilk test for normality and by Levene’s test for equality of variances. When the conditions could not be met, Kruskall-Wallis and Mann–Whitney U tests were used to compare the results between groups. The alpha level was adjusted for multiple *post hoc* comparisons of VFT characteristics between groups using the false discovery rate (FDR) method (131). Statistical significance was set at 0.05. Pearson’s correlation coefficients were computed in the ADHD groups only between VFT characteristics, clinical symptoms, and cognitive performances. Statistical analyses were performed with the Jasp© and the Jamovi© software.

## 3. Results

### 3.1. Descriptive statistics

Compared to NT controls, adults with ADHD and with ADHD + ASD reported higher depressive symptoms [χ^2^ (2) = 25.780; *p* < 0.001; *p* = 0.001] and cyclothymic traits [F (2,60) = 27.276; *p* < 0.001; *p* = 0.012, respectively]. This is also the case for anxiety in adults with ADHD [χ^2^ (2) = 18,738; *p* < 0.001]. Regarding neuropsychological measures, adults with ADHD made more errors on the Hayling Test compared to NT subjects [χ^2^ (2) = 6,708; *p* = 0.013].

### 3.2. EWFT and AVFT characteristics

**FIGURE 1:**
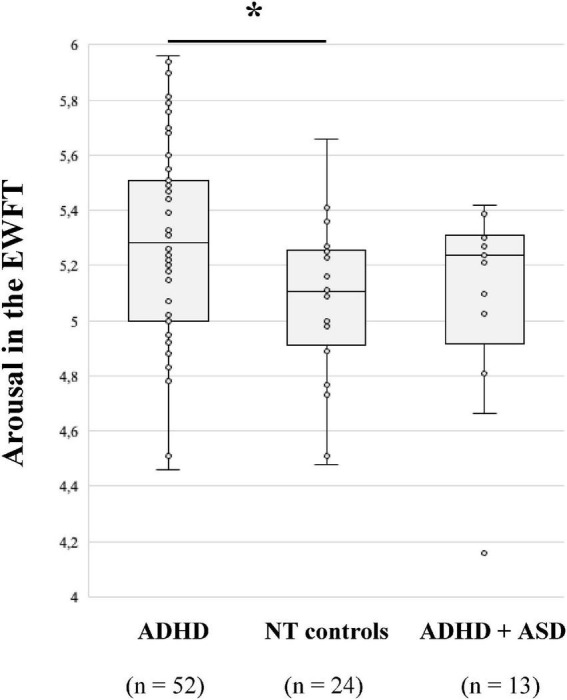
Median arousal of emotions produced in the emotion word fluency test (EWFT) according to diagnostic groups. *p = 0.033, after the false discovery rate (FDR) correction, *p* = 0.099.

First, one-way ANOVA shows that the arousal of emotions produced is significantly different between groups [F (2,86) = 4.074; p = 0.020]. *Post-hoc* test revealed that adults with ADHD generated significantly more arousing emotions (M = 5.29; *p* = 0.033; Figure 1) than NT subjects (M = 5.08). This result is no longer significant after FDR correction (*p* = 0.099). Regarding the type of emotions, adults with ADHD + ASD produced fewer anger-related words (M = 12%) compared to adults with ADHD [M = 24%; χ^2^ (2) = 8.339; *p* = 0.015 with FDR; Table 3] and NT controls (M = 24%; *p* = 0.022 with FDR). Regarding disgust and surprise the number of occurrences of related words was too rare to be analysed.

**TABLE 3:** Emotional word fluency test (EWFT) and action verb fluency test (AVFT) characteristics in each group.

	ADHD (AD) (*N* = 52)	ADHD + ASD (AS + AD) (*N* = 13)	Neurotypical controls (NT) (*N* = 24)	Statistical analyses	*P*	*Post hoc*
EWFT–number of words	7.52 (2.42)	8.15 (3.16)	8.67 (2.84)	F (2,86) = 1.598	0.208	/
EWFT–rule breaks	3.73 (3.36)	2.61 (3.15)	3.08 (2.38)	χ^2^ (2) = 2.869	0.238	/
EWFT–repetitions	0.38 (0.74)	0.15 (0.38)	0.42 (0.83)	χ^2^ (2) = 0.783	0.676	/
EWFT–frequency	0.05 (0.01)	0.05 (0.02)	0.04 (0.01)	F (2,86) = 0.469	0.628	/
EWFT–valence	4.59 (0.71)	4.64 (0.44)	4.41 (0.64)	F (2,86) = 0.715	0.492	/
EWFT–arousal	5.29 (0.35)	5.09 (0.36)	5.08 (0.28)	F (2,86) = 4.074	0.020*	AD > NT
EWFT–dominance	5.05 (0.44)	4.91 (0.23)	4.88 (0.40)	F (2,86) = 1.555	0.217	/
EWFT–% joy	35% (14%)	35% (8%)	31% (13%)	F (2,86) = 0.692	0.503	/
EWFT–% sadness	20% (12%)	27% (19%)	19% (10%)	F (2,86) = 1.800	0.653	/
EWFT–% anger	24% (15%)	12% (9%)	24% (16%)	χ^2^ (2) = 8.339	0.015**	AD, NT > AS + AD
EWFT–% fear	18% (16%)	24% (16%)	22% (14%)	χ^2^ (2) = 3.829	0.147	/
EWFT–% disgust	1% (4%)	1% (3%)	0% (2%)	χ^2^ (2) = 0.411	**/**	/
EWFT–% surprise	2% (6%)	1% (4%)	3% (6%)	χ^2^ (2) = 1.386	**/**	/
AVFT–number of words	18.22 (5.59)	14.69 (3.94)	19.54 (5.07)	F (2,85) = 3.655	0.030*	NT > AS + AD
AVFT–rule breaks	0.41 (1.47)	0.31 (0.85)	0.00 (0.00)	/	/	/
AVFT–repetitions	0.41 (0.70)	0.15 (0.38)	0.67 (1.05)	χ^2^ (2) = 2.619	0.270	/
AVFT–frequency	0.01 (0.00)	0.01 (0.00)	0.01 (0.00)	χ^2^ (2) = 2.038	0.361	/
AVFT–valence	5.76 (0.42)	5.83 (0.39)	5.84 (0.33)	F (2,85) = 0.452	0.638	/
AVFT–arousal	4.30 (0.23)	4.27 (0.26)	4.31 (0.18)	F (2,85) = 0.107	0.898	/
AVFT–dominance	5.71 (0.29)	5.80 (0.30)	5.83 (0.21)	F (2,85) = 1.670	0.194	/

*p < 0.05.

**Comparisons that remain significant after the false discovery rate (FDR) correction.

*P*-values in bold = significant *post hoc* comparisons.

Regarding the AVFT, differences between groups are significant for the number of words produced [F (2,85) = 3.655; *p* = 0.030]. Adults with ADHD + ASD produced fewer actions (M = 14.69) compared to NT subjects (M = 19.54; *p* = 0.024). However, this difference was no longer significant after the FDR correction (*p* = 0.072). The other measures, including rule breaks, repetitions, frequency, valence, arousal and dominance of generated words did not differ significantly between groups.

Secondly, we also looked at the VFT performance of a group of adults with ASD only (Table 4). Autistic adults produced more emotions (M = 10.67) and fewer rule breaks (M = 1.92) compared to subjects with ADHD (M = 7.52, *p* = 0.003; M = 3.73, *p* = 0.032, respectively; Table 4). Only the difference in the number of emotions produced remained significant after the FDR correction (*p* = 0.018).

**TABLE 4:** Comparison of demographic and neuropsychological characteristics of autistic adults (*n* = 12) with the three other groups.

	ASD (AS) (*N* = 12)	Statistical analyses	*P*	*Post hoc*
**Demographic characteristics**				
Age	26.92 (8.35)	χ^2^ (3) = 5.736	0.125	/
Gender	41.66% F	χ^2^ (3) = 3.830	0.280	/
Years of education	14.67 (1.63)	χ^2^ (3) = 6.047	0.109	/
IQ estimation	114.65 (15.82)	F (3,72) = 1.529	0.214	/
WAIS-IV–information (SS)	14.00 (2.88)	/	/	/
**Neuropsychological characteristics**				
VFT free (number of words)	54.00 (23.95)	F (3,65) = 1.171	0.021*	/
VFT letter (number of words)	23.73 (6.39)	F (3,65) = 0.465	0.087	/
VFT semantic (number of words)	33.36 (6.96)	F (3,65) = 0.893	0.072	/
EWFT–number of words	10.67 (3.31)	F (3,97) = 4.557	0.005**	AS > AD
EWFT–rule breaks	1.92 (2.19)	χ^2^ (3) = 6.432	**0.092**	AD > AS
EWFT–repetitions	0.17 (0.39)	χ^2^ (3) = 1.243	0.544	/
EWFT–frequency	0.04 (0.01)	F (3,97) = 0.698	0.556	/
EWFT–valence	4.23 (0.81)	F (3,97) = 1.247	0.297	/
EWFT–arousal	5.17 (0.16)	F (3,97) = 3.015	**0.067**	AD > NT
EWFT–dominance	4.78 (0.34)	F (3,97) = 1.960	0.125	/
EWFT–% joy	25% (14%)	F (3,97) = 1.905	0.134	/
EWFT–% sadness	19% (9%)	χ^2^ (3) = 0.987	0.804	/
EWFT–% anger	18% (11%)	χ^2^ (3) = 9.806	0.020**	AD, NT > AS + AD
EWFT–% fear	29% (12%)	χ^2^ (3) = 7.969	0.047*	AS > AD
EWFT–% disgust	7% (6%)	/	**/**	/
EWFT–% surprise	3% (5%)	/	**/**	/
AVFT–number of words	18.33 (4.72)	F (3,96) = 2.502	**0.064**	NT > AS + AD
AVFT–rule breaks	0.25 (0.87)	/	/	/
AVFT–repetitions	0.17 (0.58)	χ^2^ (3) = 4.877	0.147	/
AVFT–frequency	0.01 (0.00)	F (3,96) = 1.093	0.356	/
AVFT–valence	5.92 (0.29)	F (3,96) = 0.686	0.562	/
AVFT–arousal	4.27 (0.25)	F (3,96) = 0.116	0.951	/
AVFT–dominance	5.81 (0.20)	F (3,96) = 1.325	0.271	/

*p < 0.05.

**Comparisons that remains significant after the false discovery rate (FDR) correction.

*P*-values in bold = significant *post hoc* comparisons.

### 3.3. Correlation analyses between symptoms, EWFT and AVFT

In the ADHD group only, we were interested in the relationship between core symptoms, emotion dysregulation, cold and hot executive functions (i.e., EWFT and AVFT). Regarding ADHD symptoms, hyperactivity was related to the frequency of reported emotions (Table 5). Adults with ADHD produced more frequent emotions if they had an higher level of hyperactivity (r = 0.420; *p* = 0.012). The other main symptom domains, i.e., inattention and impulsivity, were not related to EWFT characteristics (*p* > 0.05; Table 5). Furthermore, emotional over-reactivity in adults with ADHD was associated with the number of emotions (r = 0.440; *p* = 0.008), as well as the frequency of these words in EWFT (r = –0.398; *p* = 0.018). The greater the emotional over-reactivity, the more numerous and infrequent the generated emotions were in adults with ADHD. In contrast, there was no relationship between EWFT performances and social adjustment (WRAADDS; *p* > 0.05).

**TABLE 5:** Correlation analyses between emotional word fluency test (EWFT), action verb fluency test (AVFT) and clinical characteristics in attention deficit hyperactivity disorder (ADHD) group (*n* = 52).

	WRAADDS inattention	WRAADDS hyperactivity	WRAADDS impulsivity	WRAADDS affective lability	WRAADDS emotional over-reactivity	WRAADDS social maladjustment	TEMPS-A cyclothymic temperament
EWFT–Number of words	-0.075	-0.275	-0.175	0.185	**0**.440*	-0.089	0.052
EWFT–Rule breaks	-0.002	0.169	0.052	0.061	-0.118	0.156	0.291
EWFT–Frequency	0.088	**0**.420*	0.237	-0.004	**–0**.398*	-0.103	-0.138
EWFT–Valence	-0.236	-0.076	-0.177	-0.131	0.107	-0.072	0.151
EWFT–Arousal	-0.109	-0.204	-0.229	-0.268	-0.123	-0.373	-0.395
EWFT–Dominance	-0.170	-0.072	-0.095	-0.068	0.114	-0.010	-0.115
AVFT–Number of words	-0.097	-0.056	0.019	**0**.337*	0.003	0.105	0.228
AVFT–Rule breaks	0.026	0.048	0.041	0.031	0.163	-0.059	-0.270
AVFT–Frequency	0.014	0.154	0.112	-0.171	0.082	**–0**.359*	-0.125
AVFT–Valence	-0.236	-0.076	-0.177	-0.131	0.107	-0.072	-0.067
AVFT – Arousal	0.320	**0**.387*	**0**.494*	0.287	-0.170	0.043	0.047
AVFT–Dominance	-0.122	-0.000	-0.148	-0.200	-0.043	-0.210	-0.115

*p < 0.05.

Hyperactivity (r = 0.387; *p* = 0.024; Table 5) and impulsivity symptoms (r = 0.494; *p* = 0.003) were also associated with more arousing actions on the AVFT. In addition, affective lability symptoms tended to be related to the number of actions produced (r = 0.337; *p* = 0.051). This suggests that the more adults with ADHD experience mood swings, the more actions they generate spontaneously. Social maladjustment was negatively associated with the frequency of actions reported by adults with ADHD (r = –0.359; *p* = 0.037).

### 3.4. Correlation analyses between cold executive functions, EWFT and AVFT

Concerning cold executive functions, correlation analyses indicate significant associations between performance on free, letter, semantic, and action verb VFT (*p* < 0.05; Table 6). Nevertheless, VFT measures on the free, semantic, letter and action verb conditions were unrelated to those obtained in the EWFT. With this task, increased cognitive flexibility on the TMT-B is associated with an increased number of emotions (r = –0.443; *p* < 0.008). In addition, increased inhibition errors in the Hayling test is associated with fewer emotional words generated (r = –0.337; *p* = 0.048).

**TABLE 6:** Pearsons’s correlation matrix for performance on executive tests, number of emotions and actions in attention deficit hyperactivity disorder (ADHD) group (*n* = 52).

	EWFT (number of words)	AF (number of words)	VF free (number of words)	VF semantic (number of words)	VF phono- logical (number of words)	Digit span forward (number of digits)	Digit span backward (number of digits)	Hayling test inhibition (seconds)	Hayling test inhibition (errors)	TAP Go-Nogo 1 out of 2 (errors)	TAP Go-Nogo 2 out of 5 (errors)	Stroop test inter- ferenc–reading (seconds)	TMT Part B–Part A (seconds)	ToL (total correct)	ToL (additional move- ments)	ToL (initation time)
EWFT (number of words)	–	–	–	–	–	–	–	–	–	–	–	–	–	–	–	–
AVFT (number of words)	0.236	–	–	–	–	–	–	–	–	–	–	–	–	–	–	–
VFT free (number of words)	0.049	0.613*	–	–	–	–	–	–	–	–	–	–	–	–	–	–
VFT semantic (number of words)	–0.001	0.517*	0.552*	–	–	–	–	–	–	–	–	–	–	–	–	–
VFT letter (number of words)	0.168	0.595*	0.504*	0.716*	–	–	–	–	–	–	–	–	–	–	–	–
Digit span forward (number of digits)	0.116	0.468*	0.291	0.319	0.304	–	–	–	–	–	–	–	–	–	–	–
Digit span backward (number of digits)	0.222	0.391*	0.342*	0.303	0.263	0.369*	–	–	–	–	–	–	–	–	–	–
Hayling test inhibition (seconds)	–0.108	–0.346*	–0.343*	–0.480*	–0.452*	–0.162	0.037	–	–	–	–	–	–	–	–	–
Hayling test inhibition (errors)	–0.337*	–0.232	–0.059	–0.079	–0.291	–0.050	–0.401*	0.141	–	–	–	–	–	–	–	–
TAP Go-Nogo 1 out of 2 (errors)	–0.137	–0.191	–0.103	–0.085	0.011	–0.173	–0.017	0.252	0.024	–	–	–	–	–	–	–
TAP Go-Nogo 2 out of 5 (errors)	–0.109	–0.194	–0.154	–0.056	–0.026	–0.272	0.300	0.308	–0.221	0.467*	–	–	–	–	–	–
Stroop test interference–reading (seconds)	–0.273	–0.427*	–0.351*	–0.340	–0.209	–0.344*	–0.426*	0.359*	0.131	0.032	–0.113	–	–	–	–	–
TMT Part B–Part A (seconds)	–0.443*	–0.038	0.098	–0.176	–0.004	–0.167	–0.274	0.202	0.262	0.047	–0.034	0.353*	–	–	–	–
ToL (total correct)	0.263	0.365*	0.260	0.282	0.385*	0.166	0.320	–0.154	–0.416*	–0.090	0.046	–0.032	–0.180	–	–	–
ToL (additional movements)	–0.290	–0.101	–0.065	–0.288	–0.306	–0.035	–0.306	–0.024	0.418*	0.035	–0.144	–0.020	0.165	–0.805*	–	–
ToL (initation time)	–0.193	0.336	0.165	0.413*	0.360*	0.274	0.343*	–0.167	–0.299	–0.121	–0.128	0.156	–0.201	0.428*	–0.419*	–

*p < 0.05.

For the AVFT, the faster the automatic response inhibition on the Hayling task, the more action nouns adults with ADHD can generate (r = 0.346; *p* = 0.045). The number of action verbs produced was positively related to working memory performance on the digit span task. Moreover, the number of correct responses in the ToL task, reflecting planning abilities, was also positively associated (r = 0.365; *p* = 0.034) to the number of words generated in the AVFT.

## 4. Discussion

This study is the first to use a wide range of VFT, including two conditions assessing hot executive functions, in adults with ADHD, ASD, and co-occurring ADHD + ASD, compared to NT controls. The EWFT and the AVFT conditions revealed specific patterns regarding the particularities of affective language and its potential links with emotion dysregulation symptoms in ADHD, i.e., especially emotional over-reactivity and impulsivity. Consistent with our hypotheses, adults with ADHD + ASD produced fewer action verbs compared to NT subjects. These results are congruous with those obtained by Inokuchi and Kamio in autistic adults (105) and are in line with previous studies conducted in adults with ADHD + ASD (46), indicating that the combination of these two conditions is characterized by more severe deficits compared to separate presentations of ADHD or ASD (47–49).

Indeed, process-oriented measures of the emotional output on the EWFT differed between groups. Adults with ADHD produced more arousing emotions than NT subjects. However, this result was no longer significant after FDR correction, which may be due to a lack of statistical power. Nevertheless, this result may be explained by the heightened emotional experience of adults with ADHD. Indeed, related to symptoms of emotional over-reactivity, individuals with ADHD report experiencing emotions more intensely, which could translate in the EWFT by more arousing emotional words (16–18). Consistent with Barkley’s model, which links hot executive functions, cold executive functions, and actions, it is possible that the inhibitory control impairment in ADHD may also involve emotion self-regulation abilities. Hence, inhibitory impairments in ADHD could be seen as widespread, leading to both emotional and non-emotional impulsivity (37, 42). In our study, in adults with ADHD, verbal inhibition abilities on the Hayling task were positively correlated with emotion generation in the EWFT, that is, the more people with ADHD have difficulties inhibiting a verbal response, the fewer the emotional words spontaneously generated. Given the link between emotion dysregulation and executive functions (132), it is possible that increased verbal impulsivity, especially in social interactions, is related to a lack of control over the retrieval of emotional words in ADHD, which translates here by fewer emotional words produced compared to the ASD group (69–71). This hypothesis is consistent with the functional impact of emotion dysregulation symptoms in adults with ADHD, especially in terms of social functioning (27, 133). Indeed, symptoms of ED, including emotional and verbal impulsivity, may lead adults with ADHD to say things impulsively, which can damage their social, marital, and professional relationships (27, 133). Furthermore, in past studies impulsivity in people with ADHD has been linked to increased sensation-seeking personality traits (106). As an example, heightened sensation-seeking in people with ADHD has been linked to the attraction to horror movies (104), which elicits the intense and arousing emotion of fear. Given that impulsivity was found to be positively correlated with intense actions produced in the AVFT in our ADHD group, we argue that this facet of impulsivity (i.e., sensation-seeking) in particular may be associated with intense emotional and movement-related feelings.

Regarding the emotional words related to the six primary emotions (126, 127), the ADHD + ASD group produced significantly fewer emotions related to anger, compared to NT controls and adults with ADHD. This result is consistent with studies showing deficits in the recognition of negative emotions in autistic individuals (136, 137). In particular, autistic people have deficits in the early stages of emotion recognition such as anger (97). According to Tye et al.’s (97) results and embodied cognition theories, difficulties in generating anger-related emotions are associated with the cognitive identification and processing (i.e., alexithymia) of emotions rather than a lower intensity of subjective feeling (138). Indeed, alexithymia leads to reduced emotional word production in a negative context (89). Alexithymia is frequent in ASD (139) and in ADHD (140) and appears to underlie symptoms of emotion dysregulation in autism, which are characterized by intense episodes of anger (139). Given that alexithymia and emotion dysregulation were not assessed in autistic adults in our study, the links between emotional word production, alexithymia, and emotion dysregulation would require further exploration in ASD and ADHD. This is especially important since alexithymia is related to self-harming behaviors (141, 142), used to regulate unidentified anger in ASD (31, 143).

Concerning AVFT, adults with ADHD + ASD generated fewer actions compared to NT controls. These results are consistent with those obtained by Inokuchi and Kamio (105), who found a deficit in the semantic clustering process in VFT in autistic adults compared to NT controls. A decrease in the number of action names spontaneously generated could be caused by executive dysfunction (105), motor impairment (108), or deficits in social cognition in ASD and ADHD. In addition, the performance of adults with ADHD in this task was correlated with an unconstrained VFT condition (the free condition; Table 6) which is particularly dependent on initiation strategies. Nevertheless, the difference with NT subjects was only significant in the ADHD + ASD group. These results are in line with studies postulating that the ADHD + ASD co-occurrence is not a simple addition of the cognitive alterations specific to each disorder (144). Indeed, the combination of these two conditions can lead to more severe disturbance of hot executive functions.

In ADHD, contrary to our hypothesis, participants did not produce more arousing actions compared to the other groups. Nevertheless, symptoms of hyperactivity and impulsivity were positively correlated with the generation of arousing actions. Moreover, we found a negative association between the frequency of actions generated and self-reported difficulties of social adjustment on the WRAADDS. This suggests that unusual actions are related to difficulties in social adaptation in adults with ADHD, which is consistent with the relationship between motor hyperactivity and impaired social functioning in adults with ADHD (11, 145). In our study, motor hyperactivity was also strongly correlated with emotion dysregulation and the number of actions generated was positively correlated with affective lability. Both findings are in line with those from results from past studies suggesting that emotion dysregulation is heightened in the combined ADHD subtype compared to the inattentive subtype (18, 146–149). Dysregulated arousal states have been put forward as an explanation to this association (150). It is therefore possible that dysregulated arousal states underlie affective lability in ADHD–i.e., brief and unpredictable shifts from ordinary mood to depression or mild excitation (151)—and lead to an unstable daily routine where adults with ADHD can rapidly alternate between initiated actions, akin to hyperactivity symptoms.

We also checked the EWFT and AVFT performance of autistic adults without the ADHD co-occurrence. We found that autistic adults produced significantly more emotion words compared to adults with ADHD (Table 4). Considering the observed links between the symptoms of emotion dysregulation and performance in the EWFT, these findings suggest that having increased affective language is not necessarily more adaptive, on the one hand, and that autistic adults do not have difficulties in spontaneously generating emotion words, but rather in processing and recognizing emotional states in themselves and others, on the other hand. This might also be due to the fact that people with ADHD are more impulsive, which are related to increased errors on the Hayling task, compared to autistic adults. Indeed, in an arousing context, such as the EWFT, increased verbal impulsivity might prevent people with ADHD from using efficient strategies to retrieve emotion nouns, instead of unrelated off-task words. Moreover, due to the time limited of VFT, errors prevent access to correct answers and result in decreased word output, which seems to have been the case here. This explanation is supported by the negative correlation between the number of words generated in the EWFT and verbal response inhibition errors in the ADHD group. Additionally, in our study, emotional over-reactivity in adults with ADHD was positively associated with the spontaneous generation of numerous and unusual emotions. As a matter of fact, emotional over-reactivity can lead to responses to minor stimuli in the environment that typically do not result in emotional reactions (151). Thus, adults with ADHD may have a broader repertoire of emotional experiences, both in terms of reactivity and experiences, that do not result in an increased word output in the EWFT due to their verbal impulsivity.

This study has several limitations. First, this study is limited by the small sample of adults with co-occurring ADHD + ASD and autistic adults, which results in a lack of statistical power. Analysing VFT performance in a larger sample could allow to tackle the specificities of the ASD group, including in the generation of anger-related words and action verbs. Second, we did not exclude participants with psychopathological comorbidities in the ADHD + ASD and ASD groups. These comorbidities, including anxiety and depressive disorders, are very common in these conditions (152) and are intrinsically linked to emotion dysregulation (33). In addition, hot executive functions are closely related to mood (153), and patients with mood disorders often present executive dysfunction (154–156), notably in verbal fluency (157). There also are specificities of emotion dysregulation in different presentations of ADHD—i.e., inattentive, combined and hyperactive (15). It is therefore important to explore the links between VFT performance and emotion dysregulation in different presentations of ADHD. Finally, given the potential relationship between alexithymia and sensation-seeking in spontaneous emotional and action word production in ADHD and ADHD + ASD, future studies should directly target these dimensions in relation to VFT performance in all groups.

## 5. Conclusion and perspectives

Taken together, our results suggest that even though emotional processing difficulties is a shared symptom domain between autistic adults, adults with ADHD and adults with ASD + ADHD, several differences can be found using hot executive functions conditions of VFT. First of all, adults with co-occurring ADHD + ASD could present unique affective features that are different from those found in separate presentations of ADHD or ASD, and may have more severe cognitive difficulties, especially in verbal initiation. Most importantly, these results point to the fact that their emotional symptoms do not seem to be a simple addition of both conditions. Secondly, increased emotional word output can also be dysfunctional and linked to emotion dysregulation. While future studies are needed with increased sample sizes and measures of emotion dysregulation symptoms in autistic adults, these findings suggest that different mechanisms are involved in emotional processing in each subgroup and different treatment options could be needed to target them.

## Data availability statement

The raw data supporting the conclusions of this article will be made available by the authors, without undue reservation.

## Ethics statement

The studies involving human participants were reviewed and approved by CPP South Mediterranean II. The patients/participants provided their written informed consent to participate in this study.

## Author contributions

AW: study conception, data collection and coding, statistical analysis, and write the first draft of the manuscript. EM: study conception, manuscript writing and revision, and data collection. SW: study conception, manuscript writing and revision, and data collection. LW: study conception, manuscript writing and revision, and data collection. All authors contributed to the article and approved the submitted version.
